# Bella Donna as a Palliative in Epilepsy

**Published:** 1852-07

**Authors:** M. Fredericq


From Bullet de Therap. tom. 41, p. 373.

*Belladonna as a Palliative in Epilepsy.* By M. Fredericq.

Even when this malady is not curable, it is of great importance to diminish the number of fits, which become multiplied by habit, and render the disease less and less amenable to treatment. For this purpose, M. Fredericq has advantageously used belladonna; and he gives it in the following doses to several young epileptics at the Hospital of Courtroy, reputed incurable:—Ex. Bellad., gr. iii; aquæ ℥ vi. A table-spoonful three times a day, and when premonitory symptoms are perceived.

